# Uncovering the reactive nature of 4-deoxy-l-*erythro*-5-hexoseulose uronate for the utilization of alginate, a promising marine biopolymer

**DOI:** 10.1038/s41598-019-53597-1

**Published:** 2019-11-20

**Authors:** Shota Nakata, Kousaku Murata, Wataru Hashimoto, Shigeyuki Kawai

**Affiliations:** 1 Laboratory of Basic and Applied Molecular Biotechnology, Division of Food Science and Biotechnology, Graduate School of Agriculture, Kyoto University, Uji, Kyoto 611-0011 Japan; 2 Faculty of Science and Engineering, Department of Life Science, Setsunan University, 17-8 Ikeda-Nakamachi, Neyagawa, Osaka 572-8508 Japan; 3 Laboratory for Environmental Biotechnology, Research Institute for Bioresources and Biotechnology, Ishikawa Prefectural University, 1-308 Suematsu, Nonoichi, Ishikawa 921-8836 Japan

**Keywords:** Environmental biotechnology, Metabolic engineering, Marine chemistry

## Abstract

Alginate is a linear polyuronate in brown macroalgae. It is also a promising marine biopolymer that can be degraded by exo-type alginate lyase into an unsaturated uronate that is non-enzymatically or enzymatically converted to 4-deoxy-l-*erythro*-5-hexoseulose uronate (DEH). In a bioengineered yeast *Saccharomyces cerevisiae* (DEH++) strain that utilizes DEH, DEH is not only an important physiological metabolite but also a promising carbon source for biorefinery systems. In this study, we uncovered the essential chemical nature of DEH. In particular, we showed that DEH non-enzymatically reacts with specific amino groups in Tris, ammonium salts [(NH_4_)_2_SO_4_ and NH_4_Cl], and certain amino acids (e.g., Gly, Ser, Gln, Thr, and Lys) at 30 °C and forms other compounds, one of which we tentatively named DEH-related product-1 (DRP-1). In contrast, Asn, Met, Glu, and Arg were almost inert and Ala, Pro, Leu, Ile, Phe, Val, and Asp, as well as sodium nitrate (NaNO_3_), were inert in the presence of DEH. Some of the above amino acids (Asn, Glu, Ala, Pro, Phe, and Asp) were suitable nitrogen sources for the DEH++ yeast strain, whereas ammonium salts and Ser, Gln, and Thr were poor nitrogen sources owing to their high reactivity to DEH. Nutrient-rich YP medium with 1% (w/v) Yeast extract and 2% (w/v) Tryptone, as well as 10-fold diluted YP medium, could also be effectively used as nitrogen sources. Finally, we identified DRP-1 as a 2-furancarboxylic acid and showed that it has a growth-inhibitory effect on the DEH++ yeast strain. These results show the reactive nature of DEH and suggest a basis for selecting nitrogen sources for use with DEH and alginate in biorefineries. Our results also provide insight into the physiological utilization of DEH. The environmental source of 2-furancarboxylic acid is also discussed.

## Introduction

Recently, brown macroalgae have attracted attention as a promising marine biomass for the production of biofuels and chemicals in biorefinery applications^1^. Major carbon sources from brown macroalgae are d-mannitol, laminarin, and alginate^1,2^. d-Mannitol (mannitol) is a sugar alcohol corresponding to d-mannose that can be oxidized to d-fructose by mannitol-2-dehydrogenase^3^, laminarin is a linear ß-1,3-linked glucan^4^, and alginate is a linear polyuronate consisting of mannuronic and guluronic acid residues^5^ and a promising carbon source. Alginate can be degraded by exo-type alginate lyase into an unsaturated uronate that is non-enzymatically or enzymatically converted to 4-deoxy-l-*erythro*-5-hexoseulose uronate (DEH)^6–9^; thus, DEH is an important intermediate in the physiological metabolism of alginate.

The budding yeast *Saccharomyces cerevisiae* is a well-described organism and a powerful microbial cell factory because of its robustness, safety, genetic accessibility, high tolerance to both ethanol and inhibitory compounds^10,11^. Although *S. cerevisiae* is unable to assimilate alginate, DEH, mannitol, or laminarin, three research groups, including ours, have successfully created bioengineered *S. cerevisiae* with the ability to assimilate DEH and mannitol^12–14^. Takagi *et al*. have created a bioengineered *S. cerevisiae* strain with the capacity to degrade alginate via exo-type alginate lyase expressed on the cell surface^14^. In these bioengineered yeast strains, DEH is transported into cells via a fungal DEH transporter and reduced to 2-keto-3-deoxy-d-gluconate by a bacterial reductase. Specifically, bacterial 2-keto-3-deoxy-d-gluconate kinase phosphorylates 2-keto-3-deoxy-d-gluconate to 2-keto-3-deoxy-phosphogluconate, which is cleaved into both pyruvate and glyceraldehyde-3-phosphate by bacterial 2-keto-3-deoxy-phosphogluconate aldolase^12–14^. Thus, DEH is not only an important intermediate in the physiological metabolism of alginate but also a promising carbon source for the production of biofuels and chemicals. In this study, we determined the properties of DEH that contribute to the utilization of DEH and alginate.

## Methods

### Strains, media, and plasmids

Strains and plasmids used in this study are listed in Tables 1 and 2. The prototrophic MK6286 strain was created by transformation of the autotrophic bioengineered DEH++ strain (MK5719) with plasmids carrying several autotrophic markers (Table 1). The prototrophic strain was used to avoid the addition of amino acids required by the autotrophic strain. *S. cerevisiae* strains were cultivated in yeast peptone (YP), yeast peptone dextrose adenine (YPDA), synthetic defined (SD), DEH + HN, DEH-N, DEH + Asn (5 mM), DEH + Asn (50 mM), Glc-N, Glc + Asn (5 mM), or Glc + Asn (50 mM) media. YP medium contained 1% (w/v) Yeast extract (Nacalai Tesque, Kyoto, Japan) and 2% (w/v) Tryptone (Nacalai Tesque). YPDA medium comprised YP medium with 2% (w/v) glucose and 15 mg/L adenine (pH 5.6). SD medium was a mixture of 0.67% (w/v) yeast nitrogen base without amino acids (Becton, Dickinson and Company, Franklin Lakes, NJ, USA) and with 2.0% (w/v) glucose (pH 5.6). DEH + HN medium consisted of 0.17% (w/v) yeast nitrogen base without amino acids and ammonium sulfate [AS, (NH_4_)_2_SO_4_] (Becton, Dickinson and Company), 690 mg/mL –Leu DO Supplement (Clontech, Mountain View, CA, USA), 20 mM 2-morpholinoethanesulfonic acid (MES) (pH 5.6), 1.0% (w/v) DEH, and 5 mM Asn^13^. DEH-N medium was DEH + HN medium lacking nitrogen sources (–Leu DO Supplement and Asn). DEH + Asn (5 mM) and DEH + Asn (50 mM) media were DEH-N medium supplemented with 5 mM and 50 mM Asn, respectively. Glc-N, Glc + Asn (5 mM), and Glc + Asn (50 mM) media were DEH-N, DEH + Asn (5 mM), and DEH + Asn (50 mM) media in which DEH was replaced with 2.0% (w/v) glucose. DEH-related product-1 (DRP-1) + Asn (5 mM) medium was DEH + Asn (5 mM) in which 1% (w/v) DEH was replaced with a 1% (w/v) mixture of DRP-1 and DRP-2. The 2.0% (w/v) mixture of DRP-1 and DRP-2 was prepared by incubating the reaction mixture [2.0% (w/v) DEH and 100 mM Tris-HCl (pH 7.5)] for 24 h at 30 °C, followed by sterilization by filtration through a 0.22-μm membrane. YPDA and SD media were prepared by autoclaving after mixing the components. Other media were prepared by mixing sterilized components. A 10× solution of yeast nitrogen base without amino acids and AS, and 2.5% (w/v) DEH solution were sterilized by filtration through a 0.22-μm membrane. Stock solutions of other components were autoclaved. The DEH solution was stored at −30 °C, glucose solution was stored at room temperature, and other solutions were stored at 4 °C. Media were solidified when needed by adding agar at 2% (w/v).

**Table 1:** Strains used in this study.

Strains	Description	Sources
***S. cerevisiae***		
BY4742	*MATα leu2Δ0 his3Δ1 ura3Δ0 lys2Δ0*	Euroscaf
MK5622	Autotrophic bioengineered DEH + strain; *BY4742 ade2Δ0 trp1Δ63* *P*_*TEF1*_*-yopt_eda-T*_*CYC1*_*-LEU2-P*_*TEF1*_*-yopt_kdgK-T*_*CYC1*_*/YJL219w* *P*_*TDH3*_*-yopt_DHT1-T*_*TDH3*_*-HIS3-P*_*TEF1*_*-yopt_A1-R*′*-T*_*CYC1*_*/YOL153C* *P*_*TDH3*_*-DSF1-T*_*TDH3*_*-P*_*ADH1*_*-HXT17-T*_*ADH1*_*/kanMX*	^13^
MK5719	Autotrophic bioengineered DEH++ strain = evolved MK5622 strain; *A1-R*′ c.50A > G	^13^
MK6286	Prototrophic bioengineered DEH++ strain; MK5719 pAT422, pAT424, pRS316, pRS317	This study

**Table 2:** Plasmids used in this study.

Plasmids	Descriptions	Sources
pAT422	Ap^r^ *ADE2* P_*TDH3*_ T_*TDH3*_	^27^
pAT424	Ap^r^ *TRP1* P_*TDH3*_ T_*TDH3*_	^27^
pRS316	Ap^r^ *URA3*	^28^
pRS317	Ap^r^ *LYS2*	^28^

*S. cerevisiae* strains were aerobically cultivated using a reciprocal shaker (PersonalLt-10F; Taitec, Saitama, Japan) at 30 °C and 145 strokes per minute (spm). The optical density at 600 nm (OD_600_) of the culture was measured using an Infinite 200PRO plate reader (Tecan, Männedorf, Switzerland). MK5719 stain was precultured on YPDA solid medium, whereas MK6286 was precultured on SD solid medium, suspended in sterilized pure water, and inoculated into a liquid medium to reach an OD_600_ of 0.05.

### Preparation of the DEH solution

The DEH solution was prepared and quantitated as described previously^13^, but without endo-type alginate lyase A1-I, except when stated. Briefly, an alginate solution [1% (w/v)] was prepared by autoclaving 1.0 g of sodium alginate (Nacalai Tesque) in 100 mL of pure water (Elix, Millipore). To this alginate solution, 1.0 mL of the purified exo-type alginate lyase Atu3025 (2.15 mg/mL, 8.57 U/mg), prepared as described previously^15^, was added, and the reaction mixture was incubated at 30 °C and 100 spm for 18 h. The mixture was filtered through a Centriprep-10K centrifugal filter at 4 °C and 1,600 × *g* for 30 min, and then the filtrate was freeze-dried, adjusted to a concentration of 2.5% (w/v) with pure water, and sterilized using a 0.22-μm filter.

When DEH reactivity was examined, the DEH solution with other compounds to be tested was incubated at 30 °C and the reaction stopped by immersion in ice water.

### Thin-layer chromatography (TLC)

TLC with sulfate or thiobarbituric acid staining was conducted as described previously^16^. Briefly, the reaction mixture (5.0 µL) was spotted on TLC Silica Gel 60G F_254_ (Merck, Darmstadt, Germany), which was developed using a solvent system of 1-butanol/acetic acid/water (3/2/2, v/v/v), and results were visualized by spraying the gels with 10% sulfuric acid in ethanol (sulfic acid staining) or thiobarbituric acid staining, followed by heating. Then, authenticating DEH [5.0 µL of 1% (w/v)] or 2-keto-3-deoxy-d-gluconate [1% (w/v)] was spotted on the gels.

### A1-R′ reaction

The reaction mixture [250 µL: 1% (w/v) DEH, 100 mM HEPES (2-[4-(2-hydroxyethyl)-1-piperazinyl] ethanesulfonic acid) (pH 7.5), 50 mM NADPH, and 3.0 µg (235.8 U/mg) purified A1-R′] was incubated at 30 °C, sampled sequentially for 3 h, and analyzed by TLC. The purified A1-R′ was prepared and assayed as described previously^17^. 2-Keto-3-deoxy-d-gluconate was purchased from Sigma-Aldrich (St. Louis, MO, USA).

### Isolation and structural determination of DRP-1

To determine its structure, DRP-1 was prepared by incubating 1% (w/v) DEH and 60 mM NH_4_Cl for 18 h at 30 °C. The DEH solution was prepared as described previously with endo-type alginate lyase A1-I^13^ and absorbed on 20 g of Silica Gel 60 (Nacalai Tesque). The resulting gel was dried using a desiccator and packed into a glass column. DRP-1 was eluted with a solution (300 mL) of hexane/ethyl acetate (7/3, v/v). The eluent was dried using an evaporator followed by a desiccator and solved in deuterium oxide.

The ^1^H-NMR analysis was conducted at Hitachi Power Solutions Co., Ltd. (Hitachi, Japan) on an ECA-500 FT-NMR (JEOL, Tokyo, Japan), with a magnetic field strength of 11.747 T and observation frequency range of −2.5–12.5 ppm. There were 16,384 data points, the measurement mode was non-decoupling, and the repeat time was 7 s. The cumulative number was 64 times.

GC/MS analysis was performed at Hitachi Power Solutions Co., Ltd. using a GCMS-QP2010 Plus (Shimazu, Kyoto, Japan) equipped with a capillary column (DB-5MS, 60 m × 0.25 mm I.D., df = 0.25 μm). The carrier gas was He at 180 kPa and 1.65 mL/min; the injection temperature was 300 °C; the column temperature was 50 °C (1 min), followed by a 5 °C/min increase to 300 °C (29 min); and the interface temperature was 300 °C. The injection method was split (1:20). The MS analysis was conducted at m/z 20–700 using an ion source temperature of 280 °C. Ionization was achieved using the EI method with an ionization voltage and emission current of 70 eV and 150 μA, respectively. DRP-1 in deuterium oxide was dried under nitrogen gas, resolved in ultra-pure water, and analyzed by GC/MS as described above.

## Results

### Behavior of DEH in 50 mM Tris-HCl (pH 7.5)

We recently created an autotrophic bioengineered *S. cerevisiae* DEH++ (MK5719) strain that is able to utilize DEH and mannitol^13^. During strain construction, DEH was prepared by digestion of alginate with exo-type alginate lyase Atu3025^15,16^ and, when needed, endo-type alginate lyase A1-I. The formation of DEH was verified by TLC, and DEH was visualized using the sulfate and thiobarbituric acid methods^13^. In contrast, when the exo-type alginate lyase activity of Atu3025 was assayed, DEH could be visualized using the thiobarbituric acid method but not by the sulfate method^15,16^. We attributed this difference to the Tris-HCl buffer, which was the only difference between the two systems.

**Figure 1:**
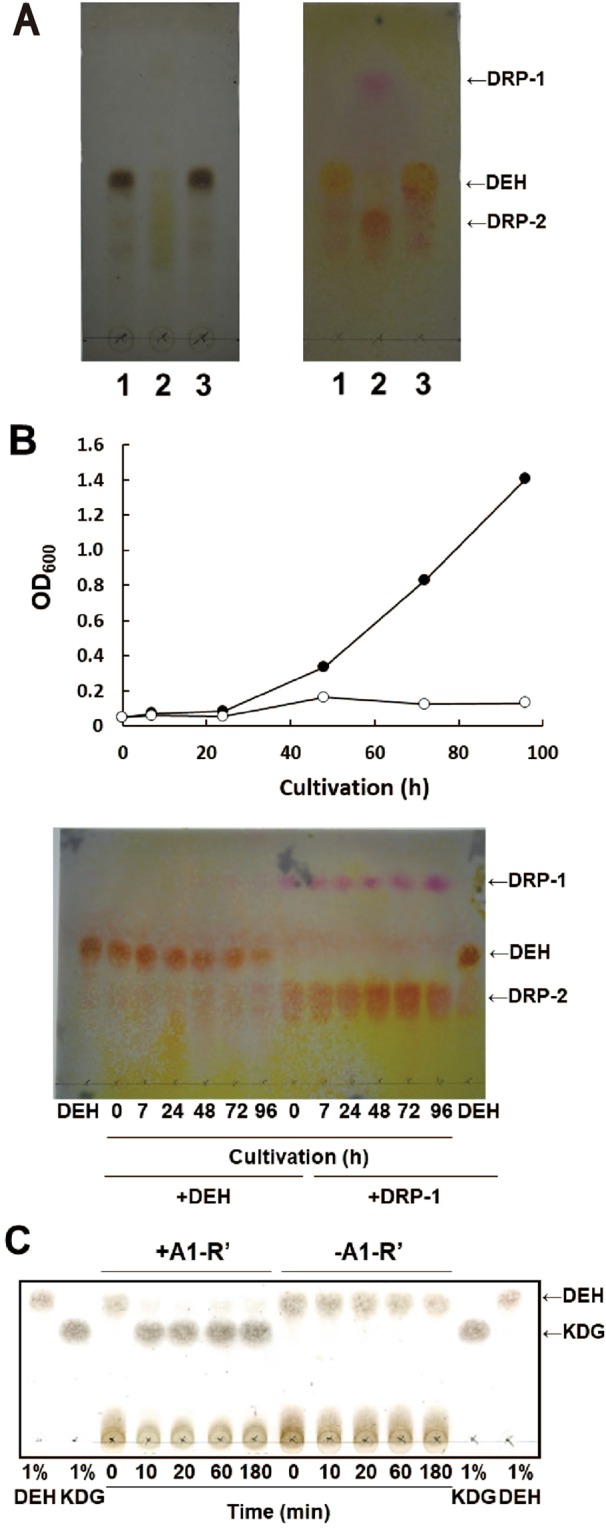
Change from DEH to DRP-1 and DRP-2. (**A**) DEH (1% w/v) changed to DRP-1 and DRP-2 after incubation with 50 mM Tris-HCl (pH 7.5). DEH (1% w/v) was incubated at 30 °C for 30 h in the presence (lane 2) or absence (lane 3) of 50 mM Tris-HCl (pH 7.5), developed, and detected using the sulfate method (left) and thiobarbituric acid (right) methods. DEH was prepared without 50 mM Tris-HCl (pH 7.5) as described previously^13^. Lane 1, 1% (w/v) DEH alone. Positions of DEH, DRP-1, and DRP-2 are indicated by arrows. (**B**) The prototrophic bioengineered *S. cerevisiae* DEH++ (MK6286) utilized DEH but not DRP-1 or DRP-2. The MK6286 strain was cultivated in 1.0 mL of DEH + Asn (5 mM) (closed symbols) or DRP-1 + Asn (5 mM) (open symbols) media (upper panel). These cultures were obtained, analyzed by TLC, and DEH was detected using the thiobarbituric acid method (lower panel). DEH corresponds to 1% DEH. The density of DEH decreased gradually in a time-dependent manner, whereas the densities of DRP-1 and DRP-2 did not decrease. We failed to determine the density of DEH because the signals were weak. (C) DEH was enzymatically reduced to 2-keto-3-deoxy-d-gluconate using purified DEH reductase (A1-R′). The reaction was conducted in the presence (+) or absence (−) of A1-R′, as described in Methods. Samples were analyzed by TLC, and DEH was detected using the sulfate method. KDG, 2-keto-3-deoxy-d-gluconate.

To confirm the effect of Tris-HCl, we prepared DEH without Tris-HCl as previously reported^13^ and then incubated the DEH with or without 50 mM Tris-HCl (pH 7.5) at 30 °C for an additional 30 h before analysis of the reaction mixtures. As expected, DEH was clearly visualized by TLC using the sulfate and thiobarbituric acid methods after incubation without 50 mM Tris-HCl (pH 7.5) (Fig. 1A). In contrast, after incubation with 50 mM Tris-HCl (pH 7.5), DEH was not detected using the sulfate method, whereas it was detected using the thiobarbituric acid method at higher and lower positions compared to that with DEH alone (Fig. 1A). We tentatively named the products DEH-related products-1 and -2 (DRP-1 and DRP-2), respectively (Fig. 1A). We observed that DEH disappeared and DRP-2 formed very quickly after DEH was mixed with Tris-HCl, but DRP-1 formed only after 18 h of incubation (Supplementary Fig. S1).

We also verified growth of the prototrophic bioengineered *S. cerevisiae* DEH++ (MK6286) strain in DEH + Asn (5 mM) medium containing DEH as a carbon source (Fig. 1B). It did not grow in DRP-1 + Asn (5 mM) medium containing a mixture of DRP-1 and DRP-2 (Fig. 1B). DEH was consumed during cultivation of this strain, while DRP-1 and DRP-2 were not (Fig. 1B). Furthermore, we found that DEH was enzymatically reduced to 2-keto-3-deoxy-d-gluconate when purified DEH reductase (A1-R′) was applied *in vitro* (Fig. 1C). Thus, we concluded that the product prepared by digestion of alginate with exo-type alginate lyase in the absence of Tris-HCl buffer was true DEH and that DRP-1 and DRP-2 were derived from DEH in 50 mM Tris-HCl (pH 7.5).

### Reactivity of DEH with amino groups

Next, we observed that, while 1% (w/v) DEH was completely transformed to DRP-1 and DRP-2 in the presence of 50 mM Tris-HCl, it was only partially changed in the presence of 10 mM Tris-HCl, irrespective of pH (pH 7.5 or 8.0) (Supplementary Fig. S2A). We noticed that 1% (w/v) DEH (molecular weight, 176) corresponded to a concentration of 56.8 mM, suggesting that 56.8 mM DEH stoichiometrically reacted with 50 mM Tris-HCl, resulting in DRP-1 and DRP-2. This hypothesis was supported by our finding that, among the tested buffer components with various pH values, DEH reacted with Tris alone and then disappeared from the chromatogram (Supplementary Fig. S2B). These findings, taken together with our previous observation that the bioengineered autotrophic DEH++ strain failed to grow in the presence of 1% (w/v) DEH plus 38 mM AS [(NH_4_)_2_SO_4_; 76 mM NH_4_^+^ ions] (Kawai, S.; unpublished data) and the fact that Tris [*tris*(hydroxymethyl)aminomethane] contains an amino group in its structure, suggested that DEH reacted with the amino group in Tris. DEH also reacted with AS, vanished, and formed DRP-1, as determined by TLC (Supplementary Fig. S2C). DRP-2 seemed to not form; thus, we focused on DRP-1 in subsequent analyses. It should be emphasized that DEH reacted only slightly with Asn, forming a very slight DRP-1 spot, and thus, Asn was almost inert in the presence of DEH (Supplementary Fig. S2C). This result was consistent with our previous results^13^ and those of another group^12^, which showed that Asn serves as a nitrogen source in the cultivation of bioengineered *S. cerevisiae* with the ability to assimilate DEH. DEH did not react with sodium nitrate (NaNO_3_), forming neither DRP-1 nor DRP-2 (Supplementary Fig. S3).

### Reactivity of DEH with amino acids

**Figure 2:**
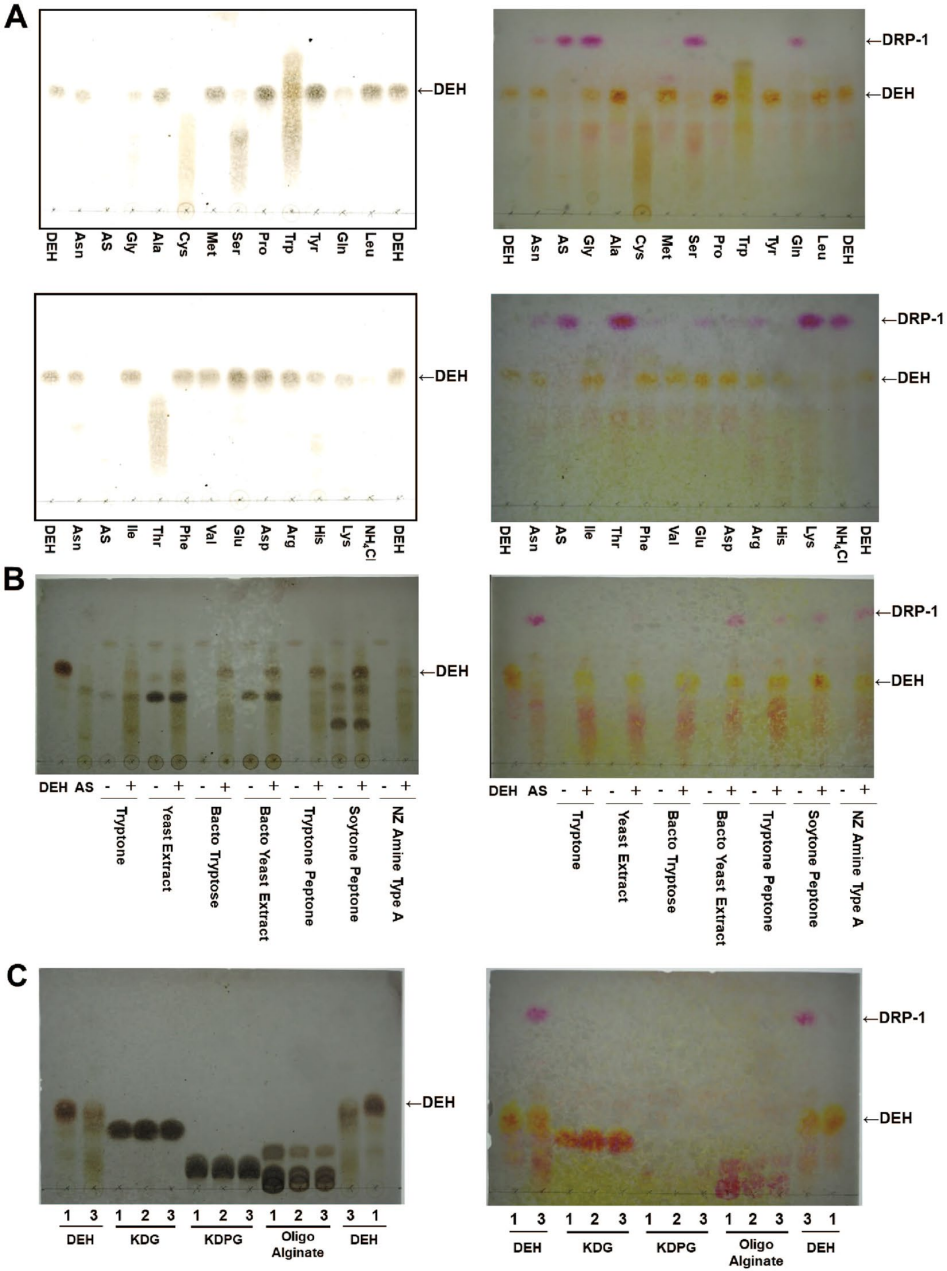
Reactivity of DEH. (**A**) Reactivity of DEH with amino acids and ammonium salts. DEH (1% w/v) was incubated in the presence of 50 mM amino acids and ammonium salts at 30 °C for 24 h, with the exception of Tyr, Trp, and Asp, which were evaluated at 1.0, 20, and 12.5 mM because of their insolubility. (**B**) Reactivity of DEH with nutrient-rich mixtures. Each of the indicated nutrient mixtures [2.0% (w/v)] or 50 mM AS [(NH_4_)_2_SO_4_] was incubated at 30 °C for 22 h in the presence (+) or absence (−) of 1% (w/v) DEH. Tryptone and Yeast extract were from Nacalai Tesque; Bacto Tryptose, Tryptone Peptone, Bact Yeast Extract, and Soytone Peptone were from Becton, Dickinson and Company; and NZ Amine Type A was from Wako. (**C**) Reactivity of NH_4_Cl with alginate metabolites. The metabolites of alginates [1% (w/v) DEH, 50 mM 2-keto-3-deoxy-d-gluconate, 50 mM 2-keto-3-deoxy-phosphogluconate, or 1% (w/v) oligoalginate] were incubated in the presence (lane 2) or absence (lane 3) of 50 mM NH_4_Cl at 30 °C for 23 h. Lane 1, authentic compounds [5.0 μL; 1% (w/v) DEH, 50 mM 2-keto-3-deoxy-d-gluconate, 50 mM 2-keto-3-deoxy-phosphogluconate, 1% (w/v) oligoalginate]. (**A–C**) Samples (5.0 µL) were spotted, developed, detected, and visualized using the sulfate (left) and thiobarbituric acid (right) methods. KDG, 2-keto-3-deoxy-d-gluconate. KDPG, 2-keto-3-deoxy-phosphogluconate.

We evaluated the reactivity of amino acids to DEH and found that DEH reacted with Gly, Ser, Gln, Thr, and Lys, clearly formed DRP-1, and then disappeared from the chromatogram; Asn, Met, Glu, and Arg were almost inert in the presence of DEH, and Asn, Glu, and Arg formed slight DRP-1 spots (Fig. 2A). We also verified that the slight spots were formed at lower positions compared to that with DRP-2, in addition to clear DRP-1 spots, when Gly and Ser reacted with DEH (Figs. 2A, S4). Moreover, DEH reacted with Cys, Trp, and His, because DEH vanished from the chromatogram after each reaction, but DRP-1 did not form (Fig. 2A). NH_4_Cl also reacted with DEH to form DRP-1, reflecting the high reactivity of ammonium ions (NH_4_^+^) (Figs. 2A, S2C). Other amino acids (Ala, Pro, Leu, Ile, Phe, Val, and Asp) were inert in the presence of DEH (Fig. 2A). Tyr did not react with DEH, although Tyr was used at a concentration of 1.0 mM owing to its low solubility, whereas others were used at 50 mM (Trp and Asp concentrations: 20 and 12.5 mM, respectively).

Furthermore, we evaluated whether ammonium salt (NH_4_Cl) reacts with metabolites of alginate (oligo-alginate, 2-keto-3-deoxy-d-gluconate, and 2-keto-3-deoxy-phosphogluconate) and confirmed that it does not; NH_4_Cl only reacted with DEH (Fig. 2C).

### Efficiency of amino acids as a nitrogen source in the presence of DEH

**Figure 3:**
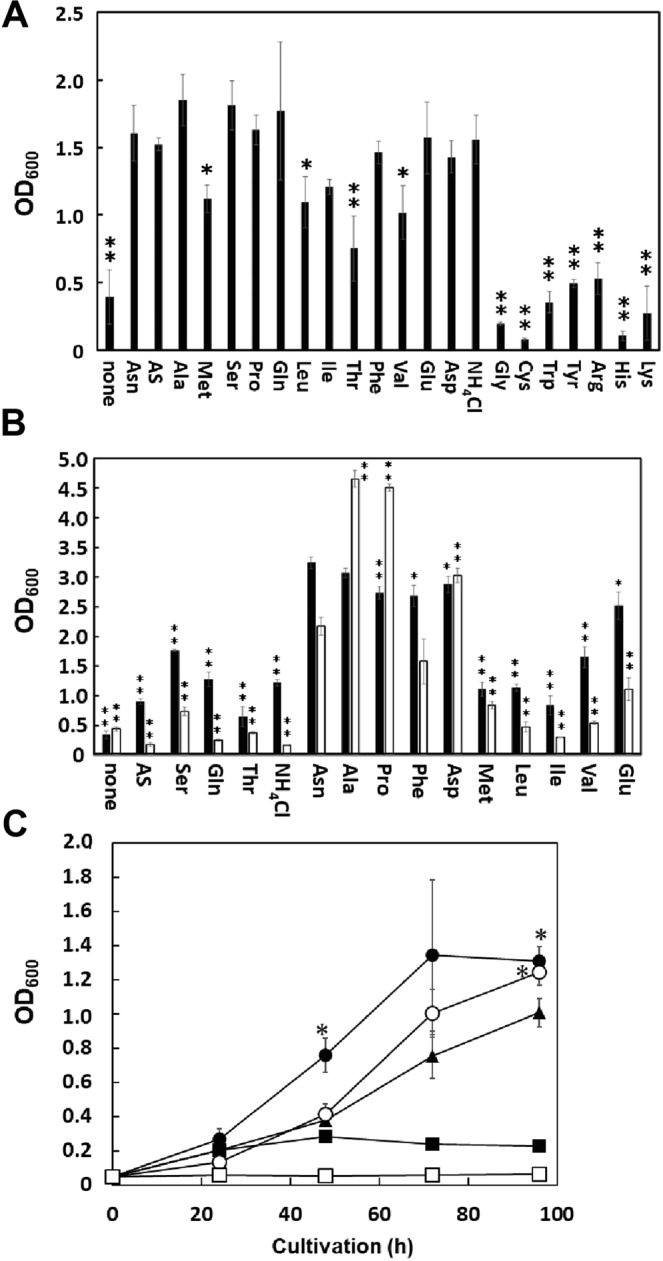
Efficiency of using amino acids, ammonium salts, and nutrient-rich mixtures as nitrogen sources. (**A**) Efficiencies of using amino acids and ammonium salts as nitrogen sources in medium containing glucose as a carbon source. Bioengineered prototrophic DEH++ (MK6286) was cultivated for 24 h in 0.25 mL of Glc-N medium supplemented with the indicated amino acids or ammonium salts at 5.0 mM or Tyr at 0.8 mM. *p < 0.05 (compared to Asn). (**B**) Efficiencies of amino acids and ammonium salts as nitrogen sources in medium containing DEH as a carbon source. The MK6286 strain was cultivated for 96 h in 0.25 mL of DEH-N medium supplemented with the indicated amino acids and ammonium salts at 5.0 mM (black bars) or 50 mM (white bars), or with Leu, Phe, or Glu at 40 mM and Asp at 10 mM (white bars). **p < 0.01, *p < 0.05 [compared to DEH + Asn (5 mM) or DEH + Asn (50 mM) medium]. (**C**) Efficiency of using nutrient-rich mixtures as nitrogen sources in medium containing DEH as a carbon source. Strain MK6286 was cultivated in 1.0 mL of YP medium (closed squares), 10-fold diluted YP medium (open squares), YP medium containing 1% (w/v) DEH (closed circles), 10-fold diluted YP medium containing 1% (w/v) DEH (open circles), and DEH + Asn (5 mM) medium (closed triangles). *p < 0.05 [compared to DEH + Asn (5 mM) medium]. (**A–C**) Averages and standard deviations are shown (n = 3).

Next, we evaluated whether each of the tested amino acids and ammonium salts could be used as a nitrogen source for the bioengineered prototrophic *S. cerevisiae* DEH++ strain in the presence of 1% (w/v) DEH. Initially, we determined that the tested amino acids and ammonium salts could serve as nitrogen sources in the presence of glucose. Gly, Cys, Trp, Arg, His, and Lys were not sufficient as sources of nitrogen at 5 mM; 0.8 mM Tyr was also insufficient (Fig. 3A). Other amino acids, such as Asn and Ala, were effectively utilized as nitrogen sources by the yeast strain (Fig. 3A). Next, we tested whether each of the amino acids [Asn, Ala, Met, Ser, Pro, Gln, Leu, Ile, Thr, Phe, Val, Glu, and Asp] and ammonium salts [AS and NH_4_Cl] could be used by the prototrophic DEH++ strain in the presence of 1% (w/v) DEH. We found that Asn, Ala, Pro, Phe, Asp, and Glu were sufficient as a nitrogen source at 5 mM; Asp was also sufficient at 10 mM; and Ala and Pro were sufficient at 50 mM (Fig. 3B). Notably, each of these amino acids was almost inert (Asn and Glu) or inert (others) in the presence of DEH (Fig. 2A). In contrast, Ser, Gln, Thr, AS, and NH_4_Cl were insufficient as nitrogen sources, especially at 50 mM (Fig. 3B), in accordance with their high reactivity (Fig. 2A). Met, Leu, Ile, and Val were also insufficient as nitrogen sources, particularly at 50 mM (and Leu at 40 mM), although they also were almost inert (Met) or inert (others) in the presence of DEH (Fig. 3B).

### Efficiency of nutrient-rich mixtures as a nitrogen source in the presence of DEH

Nutrient-rich mixtures, such as yeast extracts and tryptone, are often utilized as components in medium. We questioned whether these nutrient-rich mixtures could serve as nitrogen sources in the presence of DEH. As shown in Fig. 2B, all tested mixtures reacted with DEH, as evidenced by the decreased density of DEH shown by TLC after incubation with nutrient-rich mixtures. When DEH was incubated with Bact Yeast Extract, Tryptone Peptone, Soytone Peptone, and NZ Amine Type A, a modest amount of DRP-1 was formed; however, with Yeast extract and Tryptone, components of the YP medium, and Bacto Tryptose, no DRP-1 was produced (Fig. 2B).

Next, we evaluated whether YP medium containing 1% (w/v) Yeast extract and 2% (w/v) Tryptone could serve as a nitrogen source for the bioengineered *S. cerevisiae* DEH++ strain. As shown in Fig. 3C, YP medium was a better nitrogen source in the presence of DEH than 5 mM Asn; 10-fold diluted YP medium was also slightly superior to 5 mM Asn. This result was consistent with the results of Takagi *et al*., who used YP medium consisting of 1% (w/v) yeast extract and 2% (w/v) peptone as a nitrogen source for a bioengineered *S. cerevisiae* strain with the ability to assimilate DEH and alginate, although the sources of the yeast extract and peptone were not described^14^.

### Structure of DRP-1

**Figure 4:**
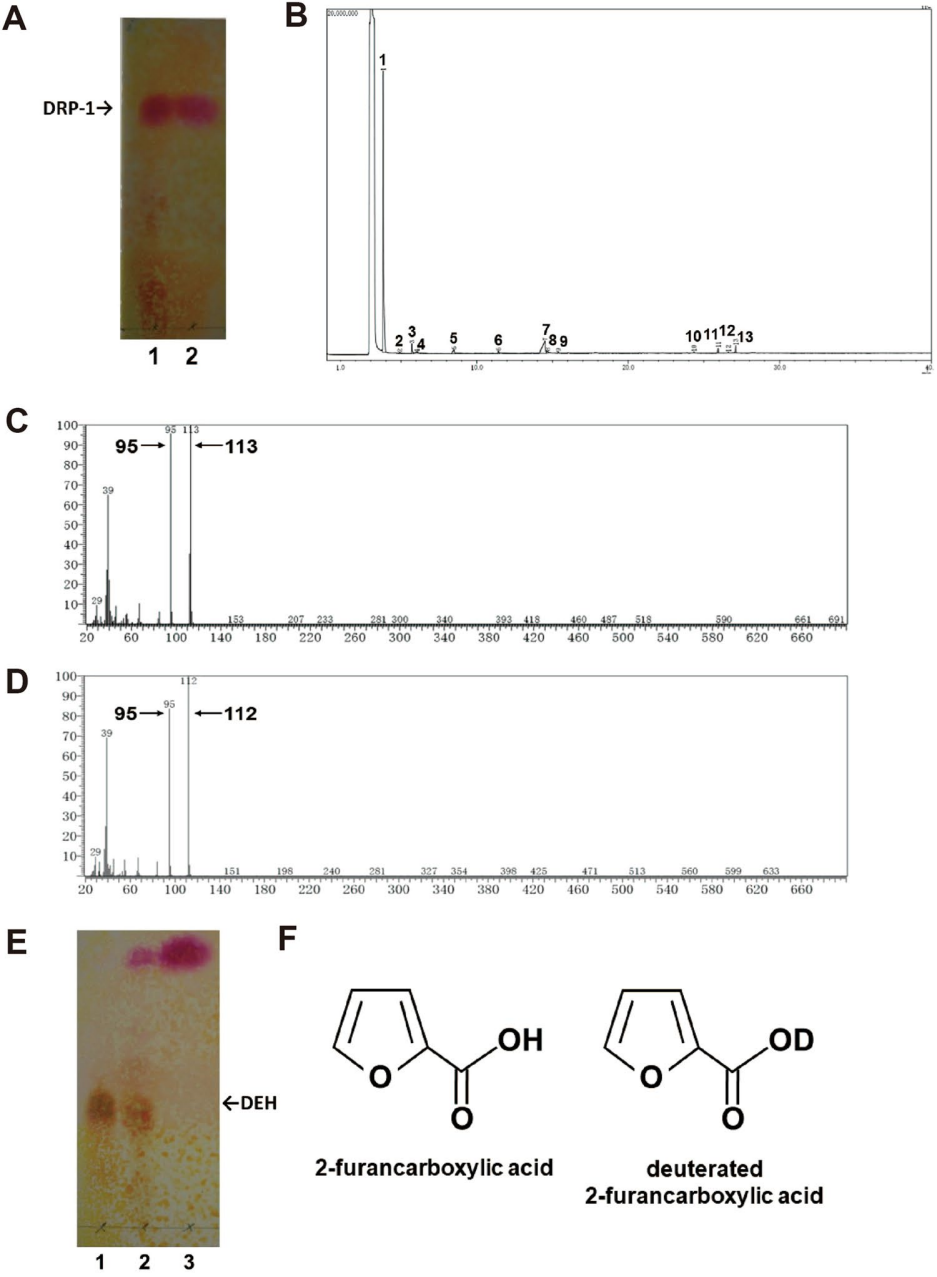
Isolation of DRP-1 and determination of its structure. (**A**) Isolation of DRP-1. Briefly, 1% (w/v) DEH was incubated in the presence of 60 mM NH_4_Cl at 30 °C for 18 h (lane 1). DRP-1 was prepared as described in Methods, isolated, and resolved in deuterium oxide (lane 2). Samples were visualized using the thiobarbituric acid method. DRP-1 is indicated by an arrow. (**B**) Total ion chromatogram obtained by GC/MS. (**C**) MS spectrum of peak 7 shown in B. (**D**) MS spectrum of DRP-1 solved in ultra-pure water. (**B–D**) GC/MS was conducted as described in the Methods. (**E**) DRP-1 was identified as 2-furancarboxylic acid. DEH was incubated in the presence of 50 mM NH_4_Cl at 30 °C for 20 h (lane 2). DEH, 1% (w/v) (lane 1); authentic 2-furancarboxylic acid, 50 mM (lane 3). (**F**) Structures of 2-furancarboxylic acid (left) and deuterated 2-furancarboxylic acid (right).

To obtain insight into the ability of DEH to react with amino groups, we isolated DRP-1 and determined its structure. DEH was incubated with NH_4_Cl, and the resulting DRP-1 was isolated using silica gel and resolved in deuterium oxide (Fig. 4A) as described in the methods section. The isolated DRP-1 was analyzed by ^1^H-NMR and GC/MS (Figs. 4B,C, S5). ^1^H-NMR signals [^1^H-NMR (500 MHz, D_2_O): δ = 6.54 (dd, *J* = 3.8 Hz and 1.8 Hz, ^1^H), 7.18 (d, *J* = 3.5 Hz, ^1^H), 7.63 (d, *J* = 1.5 Hz, ^1^H)] were detected from a 2-furancarboxylic acid derivative. Strong signals [^1^H-NMR (500 MHz, D_2_O): δ = 1.14 (t, *J* = 7.0 Hz, ^3^H), 1.98 (s, ^3^H), 4.04 (q, *J* = 7.5 Hz, ^2^H)] were also observed from ethyl acetate used to elute DRP-1 (Supplementary Fig. S5). GC chromatograms showed 13 peaks (Fig. 4B). The largest peak (no. 1) was identified as ethyl acetate by MS. Based on the MS spectrum of the 2nd largest peak (no. 7), 2-furancarboxylic acid butyl ester (molecular weight, 168) was considered a candidate compound for identification of DRP-1 (data not shown). The MS spectrum for this peak displayed signals at m/z = 113 and 95 (Fig. 4C), corresponding to deuterated 2-furancarboxylic acid (molecular weight, 113) (Fig. 4F). We assumed that a deuterated hydroxy group (-OD, molecular weight, 18) was removed from a deuterated furan carboxylic acid carrying a deuterated carboxylic acid (-COOD), resulting in a structure with a molecular weight of 95 (=113–18). To evaluate this possibility, DRP-1 in deuterium oxide was dried under nitrogen gas, resolved in ultra-pure water to replace deuterated carboxylic acid (-COOD) with carboxylic acid (-COOH), and analyzed by GC/MS. As expected, this second GC/MS analysis showed signals at m/z = 112 and 95 (Fig. 4D), suggesting that a hydroxy group (-OH, molecular mass, 17) was removed from 2-furancarboxylic acid (Fig. 4F), resulting in a structure with a molecular mass of 95 (=112–17). Thus, both ^1^H-NMR and GC/MS analyses strongly suggested that DRP-1 was 2-furancarboxylic acid. Finally, authenticated 2-furancarboxylic acid showed the same behavior as that of DRP-1 by TLC (Fig. 4E). Moreover, the Rf values of DRP-1 produced from reactions of DEH with NH_4_Cl, AS, or Tris were the same with those produced from reactions with other reactive amino acids. Thus, we concluded that DRP-1 is 2-furancarboxylic acid in all cases, which is in agreement with the fact that 2-furancarboxylic acid contains no nitrogen.

### Inhibitory effect of 2-furancarboxylic acid

Finally, we tested whether 2-furancarboxylic acid had an inhibitory effect on the bioengineered DEH++ strain, because furan-related compounds such as furfural and 5-hydroxymethylfurfural are *S. cerevisiae* fermentation inhibitors^18^, and 2-furancarboxylic acid is an inhibitor of the swarming and swimming of several environmental bacteria^19^. We observed that 2-furancarboxylic acid exhibits an inhibitory effect on growth of the DEH++ strain in Glc + Asn (5 mM) medium (Supplementary Fig. S6). Thus, the poor growth of this strain in the presence of DEH and insufficient sources of nitrogen [Ser, Gln, Thr, AS, or NH_4_Cl] (Fig. 3B) could be attributed, at least in part, to this inhibitory effect of 2-furancarboxylic acid.

## Discussion

In this study, we described the reactive nature of DEH in the utilization of alginate. In particular, we showed that DEH non-enzymatically reacts under mild conditions (at 30 °C) with amino groups in Tris, ammonium salts, and specific amino acids (e.g., Gly, Ser, Gln, Thr, Lys, Asn, Met, Glu, and Arg) and that the reaction products varied by substrate, e.g., DEH formed DRP-1 and DRP-2 when reacted with Tris (Figs. 1A,B, S2). Gln, Thr, Lys, and ammonium salts [AS and NH_4_Cl] produced clear DRP-1 spots (Fig. 2A); Gly and Ser produced the slight spots at lower positions compared to that with DRP-2, in addition to clear DRP-1 spots (Figs. 2A, S4). In contrast, Asn, Glu, and Arg formed only slight DRP-1 spots (Fig. 2A). Moreover, when DEH reacted with Tris at high pH values (pH 9.7 and pH 11.3: Supplementary Fig. S2B lanes 5 and 6), DEH did not produce DRP-1. Thus, reaction of DEH with amino groups depends on pH and the structure of molecules carrying the amino group.

**Figure 5:**
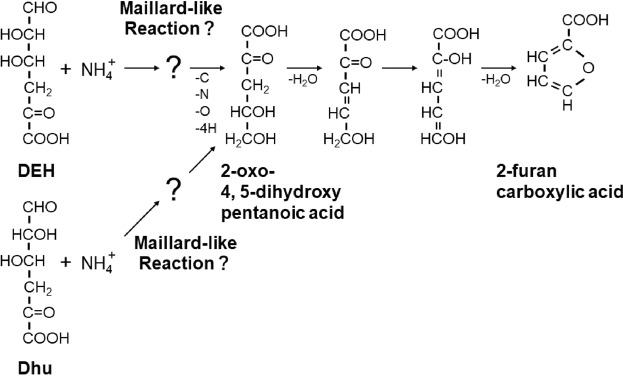
Hypothetical pathway for formation of 2-furancarboxylic acid from NH_4_^+^ and DEH or Dhu. Initially, 2-oxo-4,5-dihydroxypentanoic acid and an unidentified compound containing nitrogen are produced from NH_4_^+^ and DEH or Dhu, with a Maillard-like reaction likely triggering this pathway. Then, 2-furancarboxylic acid forms nonenzymatically from 2-oxo-4,5-dihydroxypentanoic acid. Alginate consisting of mannuronic and guluronic acid residues^5^ is degraded by exo-type alginate lyase into an unsaturated uronate that is non-enzymatically or enzymatically converted to DEH^6–9^. Dhu is formed from d-glucuronic acid residue via unsaturated glucuronic acid^26^.

The identification of DRP-1 as 2-furancarboxylic acid provides insight into how the DEH reaction proceeds. Furans can form through a Maillard reaction in which a carbonyl group initially reacts with an amino group^20^. Thus, we propose that the carbonyl group in DEH reacts with an amino group, resulting in the formation of 2-furancarboxylic acid through a Maillard-like reaction. A hypothetical pathway for 2-furancarboxylic acid formation is proposed (Fig. 5). We initially hypothesized, based on previous results^20^, that 2-furancarboxylic acid can form nonenzymatically via 2-oxo-4,5-dihydroxypentanoic acid (Fig. 5). We additionally hypothesized that, triggered by a Maillard-like reaction, DEH and NH_4_ form 2-oxo-4,5-dihydroxypentanoic acid. In this reaction, an unidentified compound containing nitrogen is produced (Fig. 5). We assume that this unidentified compound serves as a poor nitrogen source for the bioengineered DEH++ strain, because this DEH++ strain showed better growth in the presence of 5 mM NH_4_Cl than in the absence of a nitrogen source (Fig. 3B), with 5 mM NH_4_Cl able to be converted to the unidentified compounds with excess DEH.

From the view of biorefinery, this study provides an indispensable guide to selecting a nitrogen source for the use of DEH and alginate with the bioengineered DEH++ strain. When DEH reacts with certain specific amino groups, it not only degrades but also forms 2-furancarboxylic acid, which inhibits growth of the DEH++ strain (Supplementary Fig. S6). Unfortunately, ammonium salts such as AS and NH_4_Cl, which are relatively cheap, are reactive to DEH and, therefore, are not suitable for use as nitrogen sources in the presence of DEH (Figs. 2A, 3B and S2C). The amino acids that are inert or almost inert in the presence of DEH (Asn, Ala, Pro, Phe, Asp, and Glu) are considered suitable nitrogen sources for the bioengineered DEH++ strain (Fig. 3B). We also observed that YP medium consisting of 1% (w/v) Yeast extract and 2% (w/v) Tryptone and even 10-fold diluted YP medium were more effectively used as nitrogen sources than 5 mM Asn (Fig. 3C), although both Yeast extract and Tryptone reacted to DEH without producing DRP-1 (Fig. 2B). Sodium nitrate (NaNO_3_) was also inert in the presence of DEH (Supplementary Fig. S3) and, thus, is a good candidate nitrogen source. Although *S. cerevisiae* is unable to assimilate nitrate as a nitrogen source^21^, *S. cerevisiae* DEH++ strain may gain the ability to assimilate nitrate following functional expression of the nitrate utilization cluster^22^.

Sharma *et al*. reported the amino acid composition of the brown macroalga *Saccharina latissima*^23^. Based on the results^23^, an estimated 0.014 mol of reactive amino acids (Gly, Ser, Gln, Thr, and Lys) could be released from hydrolysate of 100 g [dry weight (dw)] of *S. latissima*. In addition, the brown macroalga *Laminaria digitata*, containing up to 36 g (dw) of alginate per 100 g (dw)^24^, could generate 0.18 mol of DEH following treatment with an exo-type alginate lyase^15^. Given that 0.18 mol of DEH could be generated from 100 g (dw) of *S. latissima*, 7.8% (=100 × 0.014 mol/0.18 mol) DEH could react with reactive amino acids (Gly, Ser, Gln, Thr, and Lys) and be lost. If two-step fermentation is adopted, this loss may be avoided, i.e., in the first step, the DEH++ strain ferments mannitol and consumes the endogenous reactive amino acids as a nitrogen source and, in the second step, alginate is digested to generate DEH and then the yeast ferments DEH. During this two-step fermentation, the addition of a nitrogen source can be controlled. We and other researchers conferred the ability to assimilate both DEH and mannitol on *S. cerevisiae*^12–14^, because mannitol is abundant (up to 23%) in brown macroalgae^1^.

Based on physiology and environmental microbiology frameworks, it would be reasonable that some bacteria such as *Sphingomonas* sp. A1 assimilate extracellular, environmental alginate directly without the need to extracellularly digesting alginate to DEH^2^ because there would be no risk for extracellular degradation of DEH. Unlike *Sphingomonas* sp. A1, the alginolytic marine eukaryote *Asteromyces cruciatus* can assimilate extracellular and environmental DEH but not alginate, because this marine fungus possesses a DEH transporter gene^12^. We propose that this fungus is able to assimilate extracellular and environmental DEH because low amounts of reactive ammonium salts or amino acids are present in the marine environment. In all microorganisms that metabolize DEH, intracellular DEH has the potential to react with intracellular amino groups, although not with other compounds related to alginate (oligoalginate, 2-keto-3-deoxy-d-gluconate, and 2-keto-3-deoxy-phosphogluconate) (Fig. 2C). This observation highlights the crucial role of DEH reductase in catalyzing the reduction of DEH to 2-keto-3-deoxy-d-gluconate^17^. The significance of DEH reductase is in agreement with our recent findings that the *S. cerevisiae* DEH++ strain can better metabolize DEH when DEH reductase activity is enhanced due to an E17G substitution in the introduced DEH reductase (A1-R′)^13^.

Finally, Hara *et al*. reported that agar powders include a trace amount of 2-furancarboxylic acid, which inhibits the swarming and swimming of several environmental bacteria, although the source of 2-furancarboxylic acid was not elucidated^19^. Agar is found in red macroalgae and consists of agarose and agaropectin; both are composed of repeating disaccharide units consisting of d-galactose and 3,6-anhydro-l-galactose^1^, but agaropectin also contains sulfuric acid, d-glucuronic acid, and pyruvic acid^25^. The d-glucuronic acid residue can be metabolized to 4-deoxy-l-*threo*-5-hexosulose-uronate (Dhu)^26^ by some environmental microorganisms. Thus, we propose that the 2-furancarboxylic acid in agar powder is derived from d-glucuronic acid in agaropectin, and it is possible that 2-furancarboxylic acid forms when certain amino groups react with Dhu (Fig. 5).

## Supplementary information

Suppl info

## References

[1] Kawai S, Murata K (2016). Biofuel production based on carbohydrates from both brown and red macroalgae: recent developments in key biotechnologies. Int. J. Mol. Sci..

[2] Murata K, Kawai S, Mikami B, Hashimoto W (2008). Superchannel of bacteria: biological significance and new horizons. Biosci. Biotechnol. Biochem..

[3] Horn SJ, Aasen IM, Østgaard K (2000). Production of ethanol from mannitol by *Zymobacter palmae*. J. Ind. Microbiol. Biotechnol..

[4] Myklestad, S. *Beta-1,3-glucans in diatoms and brown seaweeds*. 133–141 (Cambridge University Press, 1978).

[5] Larsen B, Salem DMSA, Sallam MAE, Mishrikey MM, Beltagy AI (2003). Characterization of the alginates from algae harvested at the Egyptian Red Sea coast. Carbohydr. Res..

[6] Hashimoto W, Miyake O, Momma K, Kawai S, Murata K (2000). Molecular identification of oligoalginate lyase of *Sphingomonas* sp. strain A1 as one of the enzymes required for complete depolymerization of alginate. J. Bacteriol..

[7] Kim HT (2012). Depolymerization of alginate into a monomeric sugar acid using Alg17C, an exo-oligoalginate lyase cloned from *Saccharophagus degradans* 2-40. Appl. Microbiol. Biotechnol..

[8] Ochiai A, Yamasaki M, Mikami B, Hashimoto W, Murata K (2010). Crystal structure of exotype alginate lyase Atu3025 from *Agrobacterium tumefaciens*. J. Biol. Chem..

[9] Hobbs JK (2016). KdgF, the missing link in the microbial metabolism of uronate sugars from pectin and alginate. Proc. Natl. Acad. Sci. USA.

[10] Hughes, S. R. & Qureshi, N. In *Biomass to biofuels: strategies for global industries* (eds Vertès, A., Qureshi, N., Yukawa, H. & Blaschek, H. P.) 55–69 (Wiley, 2010).

[11] Nielsen J, Larsson C, van Maris A, Pronk J (2013). Metabolic engineering of yeast for production of fuels and chemicals. Curr. Opin. Biotechnol..

[12] Enquist-Newman M (2014). Efficient ethanol production from brown macroalgae sugars by a synthetic yeast platform. Nature.

[13] Matsuoka F (2017). Crucial role of 4-deoxy-L-*erythro*-5-hexoseulose uronate reductase for alginate utilization revealed by adaptive evolution in engineered *Saccharomyces cerevisiae*. Sci. Rep..

[14] Takagi T (2017). Construction of bioengineered yeast platform for direct bioethanol production from alginate and mannitol. Appl. Microbiol. Biotechnol..

[15] Hirayama M, Hashimoto W, Murata K, Kawai S (2016). Comparative characterization of three bacterial exo-type alginate lyases. Int. J. Biol. Macromol..

[16] Ochiai A, Hashimoto W, Murata K (2006). A biosystem for alginate metabolism in *Agrobacterium tumefaciens* strain C58: molecular identification of Atu3025 as an exotype family PL-15 alginate lyase. Res. Microbiol..

[17] Takase R, Mikami B, Kawai S, Murata K, Hashimoto W (2014). Structure-based conversion of the coenzyme requirement of a short-chain dehydrogenase/reductase involved in bacterial alginate metabolism. J. Biol. Chem..

[18] Gorsich SW (2006). Tolerance to furfural-induced stress is associated with pentose phosphate pathway genes *ZWF1*, *GND1*, *RPE1*, and *TKL1* in *Saccharomyces cerevisiae*. Appl. Microbiol. Biotechnol..

[19] Hara S, Isoda R, Tahvanainen T, Hashidoko Y (2012). Trace amounts of furan-2-carboxylic acids determine the quality of solid agar plates for bacterial culture. Plos One.

[20] Limacher A, Kerler J, Davidek T, Schmalzried F, Blank I (2008). Formation of furan and methylfuran by maillard-type reactions in model systems and food. J. Agric. Food Chem..

[21] de Barros Pita W, Leite FC, de Souza Liberal AT, Simoes DA, de Morais MA (2011). The ability to use nitrate confers advantage to *Dekkera bruxellensis* over *S. cerevisiae* and can explain its adaptation to industrial fermentation processes. Antonie Van Leeuwenhoek.

[22] Silvestrini L (2015). Interaction of Yna1 and Yna2 is required for nuclear accumulation and transcriptional activation of the nitrate assimilation pathway in the yeast *Hansenula polymorpha*. Plos One.

[23] Sharma S (2018). Microbial protein produced from brown seaweed and spruce wood as a feed ingredient. J. Agric. Food Chem..

[24] Manns D, Nielsen MM, Bruhn A, Saake B, Meyer AS (2017). Compositional variations of brown seaweeds *Laminaria digitata* and *Saccharina latissima* in Danish waters. J. Appl. Phycol..

[25] Araki, C. In *Proceedings of the 4th International Congress of Biochemistry* 15–30 (1958).

[26] Maruyama Y (2015). Metabolic fate of unsaturated glucuronic/iduronic acids from glycosaminoglycans: molecular identification and structure determination of streptococcal isomerase and dehydrogenase. J. Biol. Chem..

[27] Ishii J (2014). Three gene expression vector sets for concurrently expressing multiple genes in *Saccharomyces cerevisiae*. FEMS Yeast Res..

[28] Christianson TW, Sikorski RS, Dante M, Shero JH, Hieter P (1992). Multifunctional yeast high-copy-number shuttle vectors. Gene.

