# Supplementary material for: Uncovering the reactive nature of 4-deoxy-l-erythro-5-hexoseulose uronate for the utilization of alginate, a promising marine biopolymer
Source: Sci Rep. 2019 Nov 20;9:17147. doi: 10.1038/s41598-019-53597-1 (PMC6868183; doi:10.1038/s41598-019-53597-1)
Supplement: Supplementary file 1 — Suppl info [file 41598_2019_53597_MOESM1_ESM.pdf]

Supplementary Figures for

Uncovering the reactive nature of 4-deoxy-L-*erythro*-5-hexoseulose uronate for the utilization of alginate, a promising marine biopolymer

Shota Nakata[1], Kousaku Murata[2], Wataru Hashimoto[1], Shigeyuki Kawai[*,3]

[1] *Laboratory of Basic and Applied Molecular Biotechnology, Division of Food Science and Biotechnology, Graduate School of Agriculture, Kyoto University, Uji, Kyoto 611-0011, Japan*

[2] *Faculty of Science and Engineering, Department of Life Science, Setsunan University, 17-8 Ikeda-Nakamachi, Neyagawa, Osaka 572-8508, Japan*

[3] *Laboratory for Environmental Biotechnology, Research Institute for Bioresources and Biotechnology, Ishikawa Prefectural University, 1-308 Suematsu, Nonoichi, Ishikawa 921-8836, Japan*

[*] Corresponding author

kawais@ishikawa-pu.ac.jp

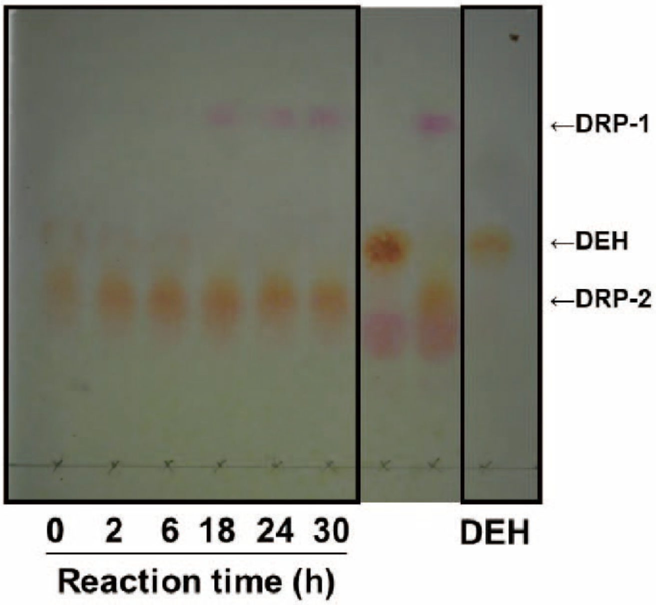

Fig. S1 DEH-Tris reaction

DEH was mixed with Tris-HCl (pH 7.5) to obtain 1% (w/v) DEH and 50 mM Tris-HCl (pH 7.5). This reaction mixture (5.0 μL) was quickly sampled and spotted on gels (0 h). The reaction mixture was incubated at 30°C for the indicated period, sampled sequentially, spotted, developed, and detected using the thiobarbituric acid method. Positions of DEH, DRP-1, and DRP-2 are indicated by arrows.

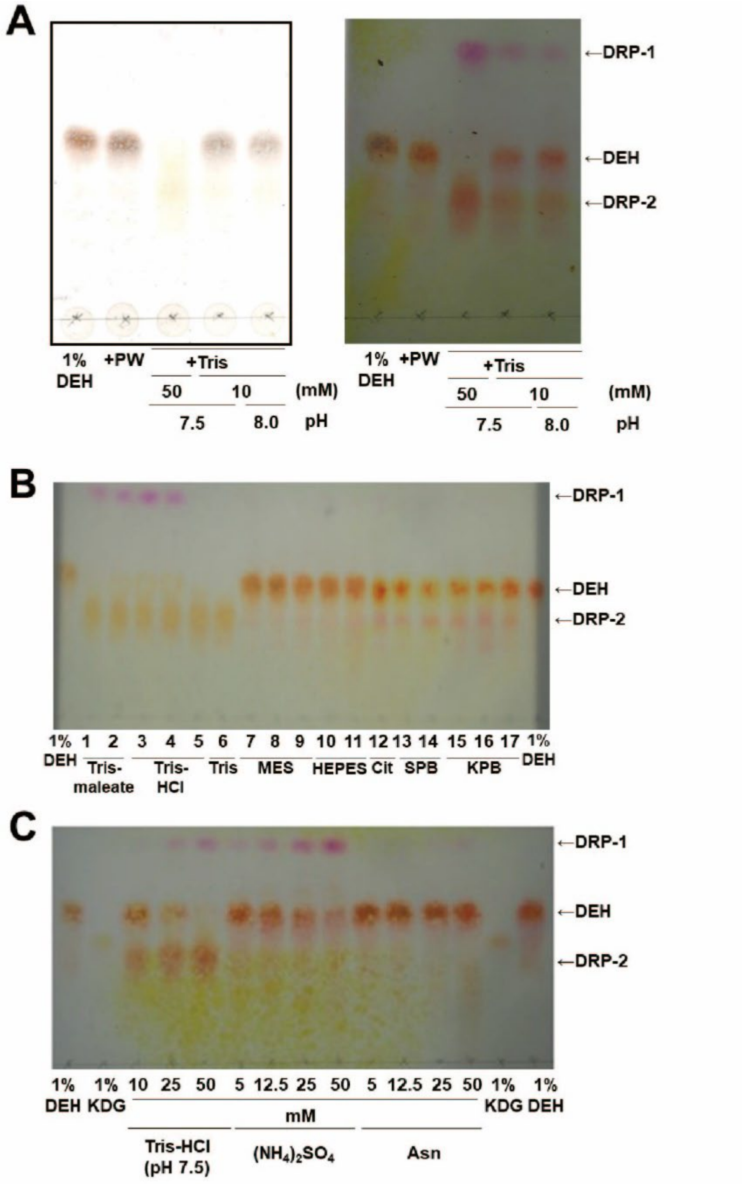

Fig. S2 DEH-Tris amino group reaction

(A) DEH (1% w/v) was incubated in the absence (+PW) or presence (+Tris) of Tris-HCl at 50 or 10 mM and at pH 7.5 or pH 8.0, as indicated. Samples were visualized using the sulfate (left) and thiobarbituric acid (right) methods. (B) DEH (1% w/v) was incubated in the presence of 50 mM buffer components, i.e., MES, MES-NaOH; HEPES, HEPES-NaOH; Cit, sodium citrate; SPB, sodium phosphate; KPB, potassium phosphate. Samples

in the indicated lanes had the following pH values: pH 6.0 (lane 1), pH 6.5 (lane 2), pH 7.5 (lane 3), pH 8.0 (lane 4), pH 9.7 (lane 5), pH 11.3 (lane 6), pH 5.6 (lane 7), pH 6.0 (lane 8), pH 7.0 (lane 9), pH 6.5 (lane 10), pH 7.5 (lane 11), pH 4.0 (lane 12), pH 6.0 (lane 13), pH 6.8 (lane 14), pH 7.4 (lane 15), pH 7.5 (lane 16), and pH 8.0 (lane 17). (C) DEH (1% w/v) was incubated in the presence of Tris-HCl (pH 7.5), $(NH_4)_2SO_4$ (AS), or Asn at the indicated concentrations. KDG, 2-keto-3-deoxy-D-gluconate.

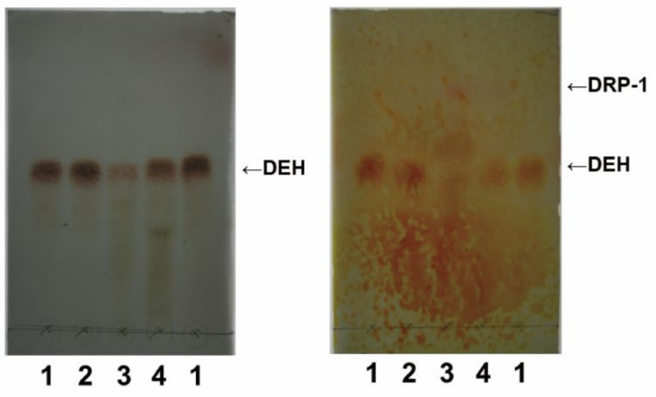

Fig. S3 DEH-sodium nitrate ($NaNO_3$) reaction

DEH (1% w/v) was incubated at 30°C for 22 h in the presence of 50 mM sodium nitrate ($NaNO_3$) (lane 2), 50 mM NH_4Cl (lane 3), or 50 mM Asn (lane 4). Samples (5.0 µL) were spotted, developed, and detected using the sulfate (left) and thiobarbituric acid (right) methods. DEH was prepared without 50 mM Tris-HCl (pH 7.5). Lane 1, 1% (w/v) DEH alone. Positions of DEH and DRP-1 are indicated by arrows.

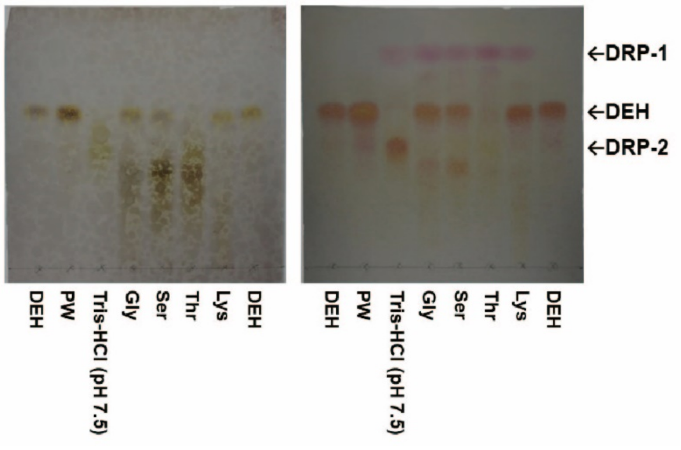

Fig. S4 DEH-Tris and DEH-reactive amino acids reaction

DEH (1% w/v) was incubated in the absence (PW) or presence of 50 mM Tris-HCl (pH 7.5) and reactive amino acids at 30°C for 24 h, as indicated.

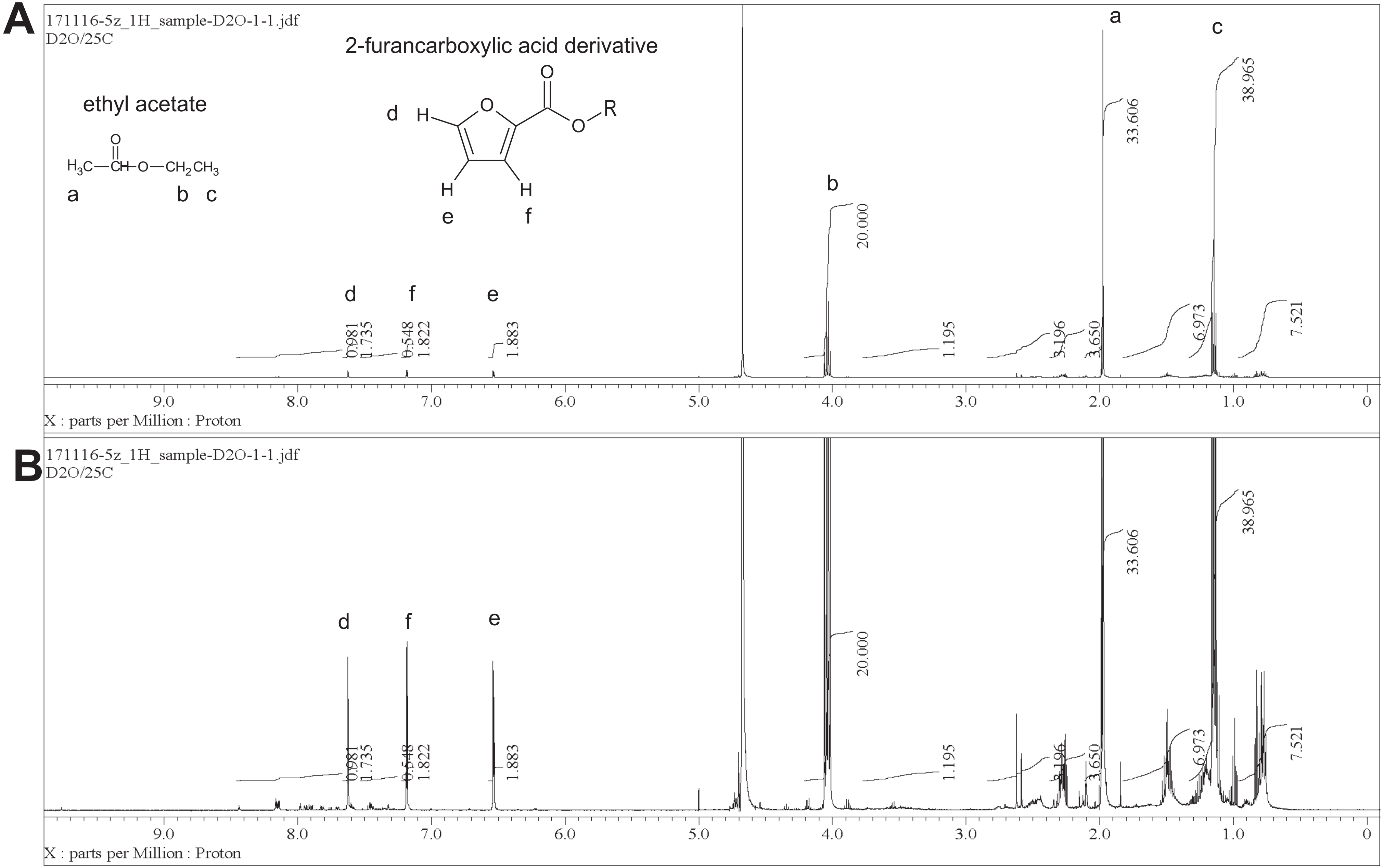
A
171116-5z_1H_sample-D2O-1-1.jdf
D2O/25C
ethyl acetate
a b c
2-furancarboxylic acid derivative
d e f
a
b
c
d
f
e
33.606
38.965
20.000
0.981
1.735
0.548
1.822
1.883
1.195
3.196
3.650
6.973
7.521
9.0 8.0 7.0 6.0 5.0 4.0 3.0 2.0 1.0 0
X : parts per Million : Proton
B
171116-5z_1H_sample-D2O-1-1.jdf
D2O/25C
d
f
e
33.606
38.965
20.000
0.981
1.735
0.548
1.822
1.883
1.195
3.196
3.650
6.973
7.521
9.0 8.0 7.0 6.0 5.0 4.0 3.0 2.0 1.0 0
X : parts per Million : Proton

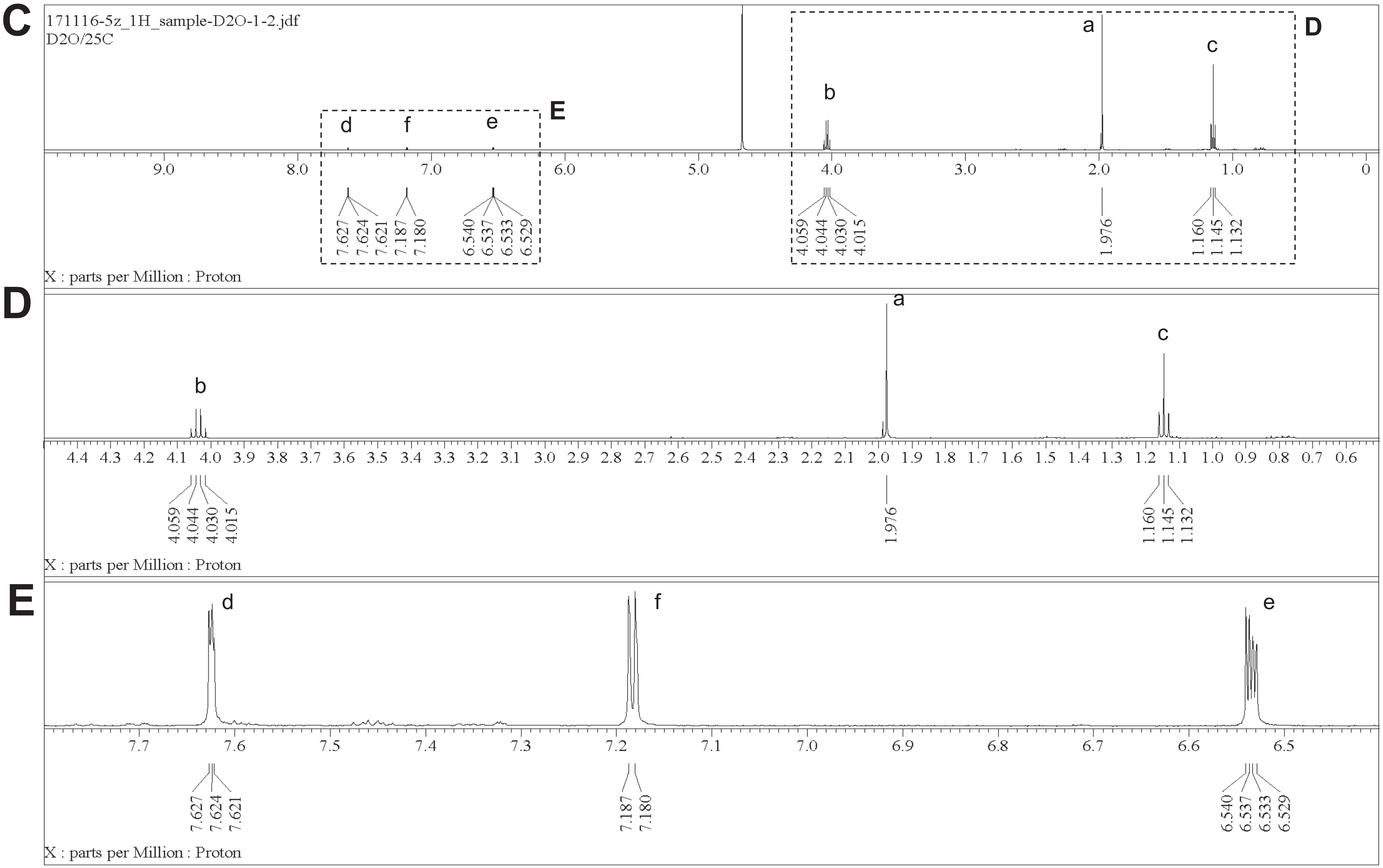
C
171116-5z_1H_sample-D2O-1-2.jdf
D2O/25C
a
b
c
d
f
e
D
E
7.627 7.624 7.621 7.187 7.180 6.540 6.537 6.533 6.529
4.059 4.044 4.030 4.015
1.976
1.160 1.145 1.132
X : parts per Million : Proton
X : parts per Million : Proton
X : parts per Million : Proton

Fig. S5 Summary of ^{1}H-NMR results for DRP-1 showing signals consistent with a 2-furancarboxylic acid derivative (d–f) and ethyl acetate (a–c)

B shows data from A expanded on the vertical axis, and D and E are extensions of C, as indicated.

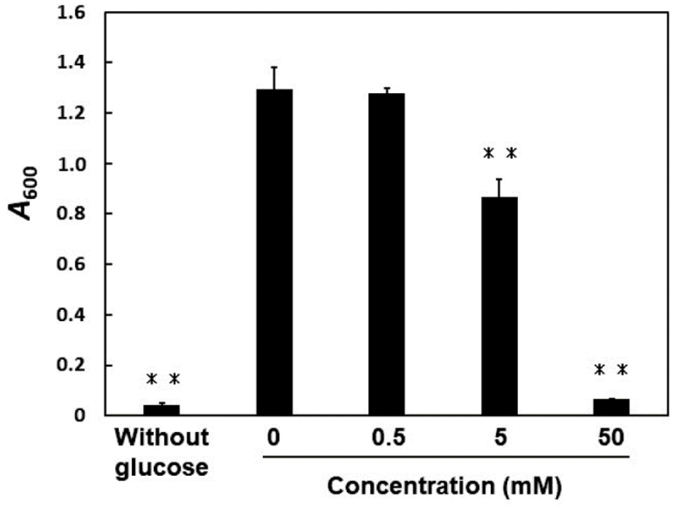

Fig. S6 Effect of 2-furancarboxylic acid on growth of the bioengineered DEH++ strain

The prototrophic bioengineered *S. cerevisiae* DEH++ strain (MK6286) was cultivated for 24 h in 1 mL of Glc+Asn (5 mM) medium containing the indicated concentrations of 2-furancarboxylic acid or Glc+Asn (5 mM) medium lacking glucose. **$p < 0.01$ (compared to 0 mM 2-furancarboxylic acid). Averages and standard deviations are shown (n = 3).
